# Antigen-experienced NK-T cells: integrating new insights and exploring therapeutic potential in cancer immunotherapy

**DOI:** 10.3389/fimmu.2026.1877232

**Published:** 2026-09-22

**Authors:** Shunichiro Kuramitsu, Masasuke Ohno, Mitsugu Fujita

**Affiliations:** 1 Department of Neurosurgery, Shizuoka Cancer Center, Nagaizumi, Japan; 2 Department of Neurosurgery, Nagoya University School of Medicine, Nagoya, Japan; 3 Department of Neurosurgery, Aichi Cancer Center, Nagoya, Japan; 4 Center for Medical Education and Clinical Training, Kindai University Faculty of Medicine, Sakai, Japan

**Keywords:** antigen-experienced NK-T (AENK-T) cells, chimeric antigen receptor (CAR)-T cells, immune checkpoint blockade, innate-like T cells, natural killer cell receptors, T cell exhaustion, tumor immune microenvironment (TIME)

## Abstract

Conventional CD8+ T cells can acquire natural killer (NK) cell receptors upon persistent antigen exposure, forming antigen-experienced NK-T (AENK-T) cells. Their dual-state plasticity within the tumor immune microenvironment determines cancer immunotherapy outcomes. This narrative review synthesizes recent transcriptomic, epigenomic, and functional data to define the trajectory, regulation, and therapeutic relevance of AENK-T cells. The analysis establishes a “temporal hierarchy” differentiating AENK-T cells from innate-like T cells: activating NK receptors (NKG2C, NKG2D) emerge early, while inhibitory receptors (NKG2A, killer-cell immunoglobulin-like receptors, KLRB1) accumulate under sustained stimulation. Mechanistically, BCL11B downregulation and the T-bet–Zeb2 axis stabilize a cytotoxic Effector AENK-T state. Conversely, the TOX–LAG-3 loop and SOX4–ID3 axis drive transition into a suppressive Exhausted AENK-T state, mirroring terminal exhaustion. The tumor immune microenvironment accelerates this shift via hypoxia, metabolic competition, and TGF-β, contrasting with chronic infection models. Translating these findings, an evaluation of emerging strategies, such as NKG2A blockade, biomarker-guided patient selection, and next-generation chimeric antigen receptor (CAR)-T cell engineering, suggests that temporally informed approaches targeting the AENK-T trajectory may overcome current immune checkpoint therapy limitations.

## 1 Introduction

The immune system is traditionally divided into innate and adaptive branches. Adaptive immunity is characterized by T cells expressing antigen-specific T cell receptors (TCRs) and B cells expressing antigen-specific B cell receptors (BCRs). In contrast, innate lymphoid cells, such as natural killer (NK) cells, have been defined by their exclusive definition that they are “neither T cells nor B cells.” (1). However, advanced single-cell technologies have revealed a more complex landscape within the tumor immune microenvironment (TIME) (2). T cells and NK cells share common lymphoid progenitors. Several “innate-like T cell” populations bridge these two branches by possessing both TCRs and rapid innate-like cytotoxicity (3, 4). Examples include invariant NKT (iNKT) cells, mucosal-associated invariant T (MAIT) cells, gamma delta (γδ) T cells, and innate-like T cells with high cytotoxic potential (ILTCKs) (5, 6). These populations represent developmentally programmed lineages that mature primarily in the thymus. Unlike these distinct lineages, accumulating evidence demonstrates that conventional post-thymic TCRαβ+ T cells can acquire NK cell-associated receptors and transcriptional programs peripherally. This phenotypic shift occurs following persistent antigen experience, notably during chronic stimulation in infections or within the TIME. Because this convergence blurs the boundary with populations such as iNKT cells, these peripheral T cells have been inconsistently termed NKT, NK-like, or innate-like T cells.

To distinguish them from thymically-derived innate-like T cells, this review uses the term “antigen-experienced NK (AENK)-T cells.” This designation refers to conventional TCRαβ+ T cells that functionally acquire NK cell-associated programs following antigen exposure. Because AENK-T cells and innate-like T cells converge on nearly identical NK receptor (NKR) expression profiles and functional programs, distinguishing them by single surface markers remains a technical challenge. Accurate delineation currently relies on tracking developmental trajectories via single-cell RNA sequencing (scRNA-seq) or identifying remnants of diverse, conventional TCRαβ repertoires (4). While various T cell subsets, including CD4+ T cells, can display NKRs such as KIRs and NKG2C upon chronic stimulation (7, 8), this review focuses on CD8+ T cells. This focus is due to their primary role in direct tumor cytotoxicity and their profound interconnection with canonical exhaustion lineages within the TIME.

Initially, the AENK-T transition is linked to the acquisition of potent effector functions and enhanced cytotoxicity (9). However, under chronic antigen stimulation within the TIME, this trajectory bifurcates, becoming an integral part of a developmental path toward terminal exhaustion. Thus, AENK-T cells exhibit a dual nature: potent innate-like cytotoxicity alongside a vulnerability to exhaustion. Clarifying this context-dependent plasticity facilitates the design of immunotherapeutic strategies that harness the effector functions of AENK-T cells while mitigating their exhaustion.

## 2 AENK-T cell differentiation as a specialized branch of T cell exhaustion

As described above, the peripheral differentiation of AENK-T cells is fundamentally intertwined with the process of T cell exhaustion. This progression establishes a “temporal hierarchy.” We define this hierarchy as the chronological acquisition and loss of specific NKRs. In this stage-wise process, activating receptors are typically induced early, while inhibitory receptors accumulate later. This sequence is driven by the duration and intensity of continuous antigen exposure.

Upon acute antigenic stimulation, naïve T cells (Tnaive) undergo rapid clonal expansion and differentiate into short-lived effector cells (SLECs) and memory precursor effector cells (MPECs) (10). Conversely, chronic antigenic stimulation within environments like the TIME induces a progressively dysfunctional state termed T cell exhaustion (11). The canonical exhaustion trajectory proceeds sequentially: Tnaive differentiate into progenitor exhausted T cells (Tex^prog^), then intermediate exhausted T cells (Tex^int^), and ultimately into terminally exhausted T cells (Tex^term^) (**Figure 1**) (12, 13).

**Figure 1:**
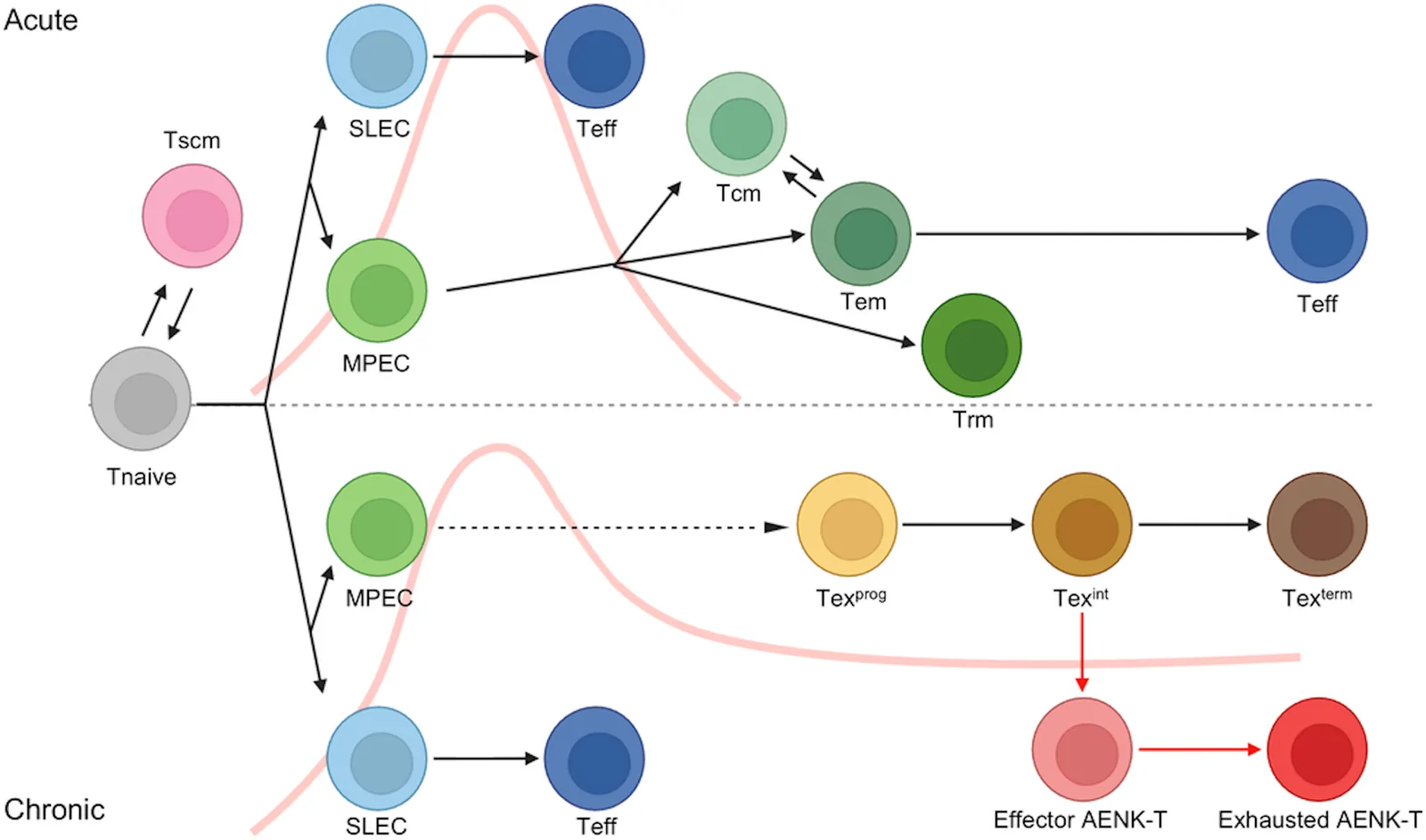
T cell differentiation pathways under acute and chronic antigen stimulation. The distinct differentiation trajectories of T cells following acute (upper panel) versus chronic (lower panel) antigen exposure are illustrated. Acute stimulation: Naive T cells (Tnaive) differentiate into short-lived effector cells (SLEC) and memory precursor effector cells (MPEC). MPECs can further develop into various memory T cell subsets (Tscm, Tcm, Tem, and Trm), which contribute to the “Memory pool”. SLECs primarily become effector T cells (Teff). Chronic stimulation: Tnaive cells can also give rise to SLECs and Teffs. However, persistent antigen exposure leads MPECs towards a “Proliferation/Stem-like” state. These cells differentiate into progenitor (Tex^prog^) and intermediate (Tex^int^) exhausted T cells, and ultimately give rise to terminally exhausted T cells (Tex^term^). Highly cytotoxic Effector AENK-T cells diverge from the canonical path at the Tex^int^ stage. The divergence is driven by a complex interplay of sub-optimal TCR avidity, costimulatory availability, and microenvironmental cytokine cues. Under sustained chronic stimulation, these cells transit into dysfunctional Exhausted AENK-T cells.

AENK-T cells do not completely deviate from the exhaustion pathway. Instead, their early states represent a highly functional metabolic and phenotypic divergence. Single-cell multiomic analyses with paired TCR sequencing place this divergence at the Tex^int^ stage rather than upstream of it. Late-stage exhausted cells resolve into a terminally exhausted subset and a cytotoxic, NK gene-expressing subset; adoptive transfer of sorted Tex^int^ cells gives rise to both, identifying Tex^int^ as a branch point (14). Individual clones are biased toward one fate or acquire both, where low TCR signaling avidity correlates with the cytotoxic NK-like fate and high avidity correlates with terminal exhaustion (14). Pseudotime analyses of longitudinal single-cell data support this branch from Tex^int^ and demonstrate that the acquisition of NK-lineage programs depends on the transcription factor Zeb2 (15). These cytotoxic NK gene-expressing T cells generated within the canonical exhaustion trajectory correspond to Effector AENK-T cells. Costimulatory availability and microenvironmental cytokine cues act alongside this signal-strength effect, and cells that adopt the highly cytotoxic Effector AENK-T phenotype extend their functional window. The extent to which Tex^int^-independent routes contribute remains undetermined. However, following the temporal hierarchy, this state is restricted. Under sustained metabolic stress (e.g., hypoxia) and suppressive pressures within the tumor parenchyma, these cells are thought to continuously acquire inhibitory NKRs. They eventually transition into the Exhausted AENK-T state, which we define operationally by the high expression of inhibitory NKRs. This state functionally overlaps with canonical Tex^term^ cells, in that both lose effector function; whether the two converge phenotypically remains undetermined.

The spatial context further delineates this branching trajectory. In systemic immune responses, the anatomic sites are better defined. Tumor-specific CD8+ T cells in tumor-draining lymph nodes (TDLNs) retain a stem-like TCF1+ phenotype, are the clonal precursors of their more differentiated intratumoral counterparts and replenish the tumor through continuous migration (16). An intermediate exhausted population in the white pulp of the spleen expands under immune checkpoint blockade and gives rise to tumor-infiltrating clonotypes, and the amount of systemic antigen determines how far this population is redirected toward a circulating Effector AENK-T state (17) (**Figure 2**). High antigen density within these peripheral lymphoid organs therefore diverts part of the trajectory toward the functionally competent Effector AENK-T state. The same study reports that the corresponding Effector AENK-T population in human blood has a diminished capacity to traffic into tumors, which suggests that this diversion trades tumor infiltration for cytotoxic competence.

**Figure 2:**
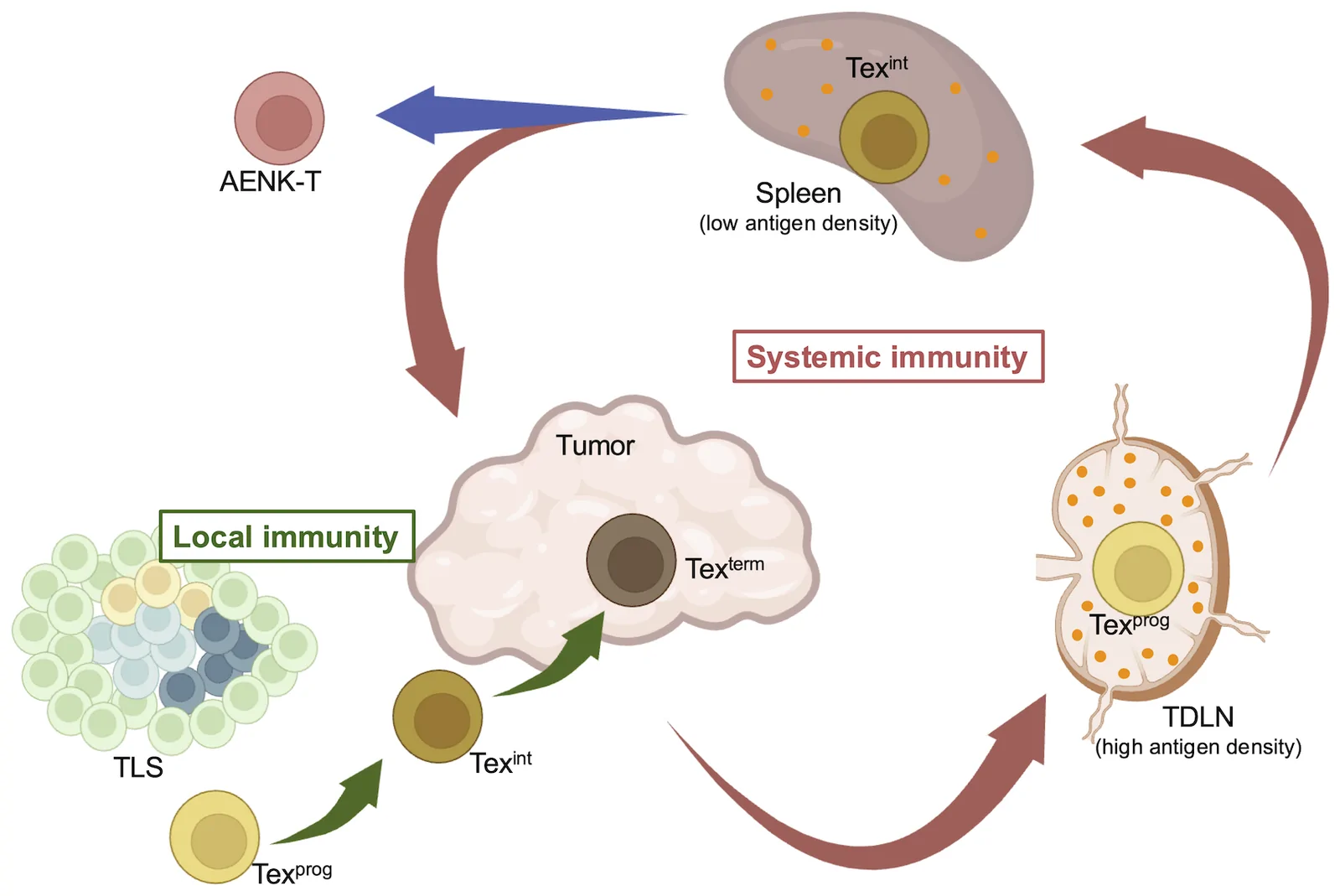
Systemic and local differentiation trajectories of AENK-T cells. Systemic immune responses (red circular arrows): T cells primed in tumor-draining lymph nodes (TDLNs) migrate as progenitor exhausted T cells (Tex^prog^) and differentiate into intermediate exhausted T cells (Tex^int^) in the spleen. Within these peripheral pools, high local antigen density diverts the trajectory toward a functionally competent Effector AENK-T state. Local immune responses within the tumor immune microenvironment (TIME) (green arrows): Tertiary lymphoid structures (TLS) sustain local Tex^prog^ pools. Continuous antigen exposures and microenvironmental factors drive the transition into terminally exhausted (Tex^term^) phenotypes.

In contrast, the developmental dynamics within the localized TIME remain poorly understood. While tertiary lymphoid structures (TLS) support localized Tex^prog^ pools (18, 19), the specific mechanisms governing local AENK-T cell development remain undefined. Evidence suggests that T cells infiltrating deep into the tumor-associated stroma and parenchyma encounter harsh microenvironmental pressures within the tumor core, driving Effector AENK-T cells into terminal exhaustion and transitioning them into the immunoregulatory Exhausted AENK-T (and Tex^term^) phenotype (Section 4.3).

## 3 AENK-T cells in biological contexts: translating infection models to the tumor immune microenvironment

The physiological framework for T cell exhaustion was originally characterized in chronic viral infections before being mapped onto cancer immunology (20, 21). Although this review focuses on the TIME, infection models provide a crucial physiological baseline for AENK-T cells. Comparing these contexts highlights how the tumor environment dysregulates the temporal hierarchy. In cancer, this leads to anomalous pathological exhaustion instead of a balanced host defense.

### 3.1 The physiological paradigm vs. pathological exhaustion in infection

Infections impose persistent antigen stimulation that drives AENK-T cell emergence. However, the outcome depends heavily on the infection dynamics. A longitudinal analysis of primary human CMV infection has shown that NKR induction on CD8+ T cells begins in the acute phase and is largely sustained: activating and inhibitory receptors are induced to a similar extent, and 19 of the 29 induced receptors remain high one year after viral replication ceases. Within this response the order is preserved, since activating CD94^dim^NKG2C dimers reach the cell surface before inhibitory CD94^bright^NKG2A dimers (22). This is direct human *in vivo* evidence for the temporal hierarchy.

Prototypical chronic infections like CMV deliver persistent stimulation through non-classical MHC class I ligands (e.g., HLA-E presenting UL40-derived peptides). The CMV-induced AENK-T cells that emerge under this stimulation maintain high cytotoxicity and low PD-1 expression, successfully navigating chronic stimulation without falling into profound dysfunction (23, 24). Similar robust Effector AENK-T responses are observed in non-viral chronic infections like leprosy (9). This creates a “healthy” version of the temporal hierarchy.

However, it is an oversimplification to equate all infections with healthy AENK-T cells and tumors with exhausted ones. Severe chronic viral infections (such as Hepatitis B or C) drive T cells into classical exhaustion that closely mirrors the TIME. This results in diminished effector capacities and a high inhibitory receptor load (21, 25, 26).

### 3.2 Cancer and the TIME as a dysfunctional niche

In contrast to the controlled stimulation of viral infections, the TIME is characterized by heterogeneous and continuous antigen exposure coupled with severe microenvironmental constraints (27). Chronic infection imposes persistent TCR stimulation within relatively permissive systemic tissues. Conversely, the TIME combines antigen persistence with severe metabolic stress (hypoxia, nutrient depletion, and acidosis) (28), soluble suppressive mediators (such as TGF-β), and dense expression of inhibitory ligands (HLA-E, HLA-C, and CLEC2D) on tumor cells. This unique microenvironmental combination, rather than antigen persistence alone, alters the temporal hierarchy and accelerates the transition of AENK-T cells into terminal exhaustion (29).

Chimeric antigen receptor (CAR)-T cell models demonstrate that activating NKRs like NKG2C are only transiently expressed during the early phases of antigen exposure in the TIME. These receptors subsequently decline, while NKG2A and KLRB1 progressively upregulates in synchrony with classical exhaustion markers (30). **Table 1** summarizes the molecules tracked across this transition, including NKRs and classical checkpoint receptors, along with their temporal expression dynamics and clinical prognostic significance, and **Figure 3** shows the corresponding protein-level time course. The classical checkpoint receptors PD-1 and CTLA-4 exhibit an early transient upregulation followed by a decline to moderate levels (similar to NKG2C), whereas TIGIT progressively increases in parallel with the inhibitory NKRs. TIM-3 is also higher after chronic stimulation, but the available data compare two time points only, so whether it follows the early transient pattern of NKG2C or the progressive pattern of NKG2A remains undetermined.

**Table 1:** NK cell receptors (NKRs) and classical checkpoint receptors tracked during AENK-T cell differentiation.

Molecule (*gene*)	Type	Ligand	AENK-T state	Temporal expression (30)	Clinical prognosis	Key references
NKG2D (*KLRK1*)	Activating NKR	MICA/B	Effector	—	Favorable	(31–33)
NKG2C (*KLRC2*)	Activating NKR	HLA-E	Effector	Early transient	Not established	(24, 30, 34)
NKG2A (*KLRC1*)	Inhibitory NKR	HLA-E	Exhausted	Late progressive increase	Poor	(30, 35, 36)
Inhibitory KIR (*KIR2DL1/2*)	Inhibitory NKR	HLA-C	Exhausted	Increase	Poor	(30, 37)
KLRB1 (*KLRB1*)	Inhibitory NKR	CLEC2D (LLT1)	Exhausted	Late progressive increase	Context-dependent	(30, 38, 39)
PD-1 (*PDCD1*)	Classical Checkpoint	PD-L1/ PD-L2	—	Induced early, then declines to moderate level	Poor	(30)
CTLA-4 (*CTLA4*)	Classical Checkpoint	CD80/CD86	—	Induced early, then declines to moderate level	Poor	(30)
TIM-3 (*HAVCR2*)	Classical Checkpoint	Galectin-9/CEACAM1	—	Increase	Poor	(30)
TIGIT (*TIGIT*)	Classical Checkpoint	CD155/CD112	—	Late progressive increase	Poor	(30)

Each molecule (NKR or classical checkpoint receptor) is classified by type/category, cognate ligand, and associated AENK-T differentiation state. The table also indicates the temporal expression pattern during chronic stimulation (based on CAR-T models (30)), clinical prognostic significance across solid tumor types, and key references.

**Figure 3:**
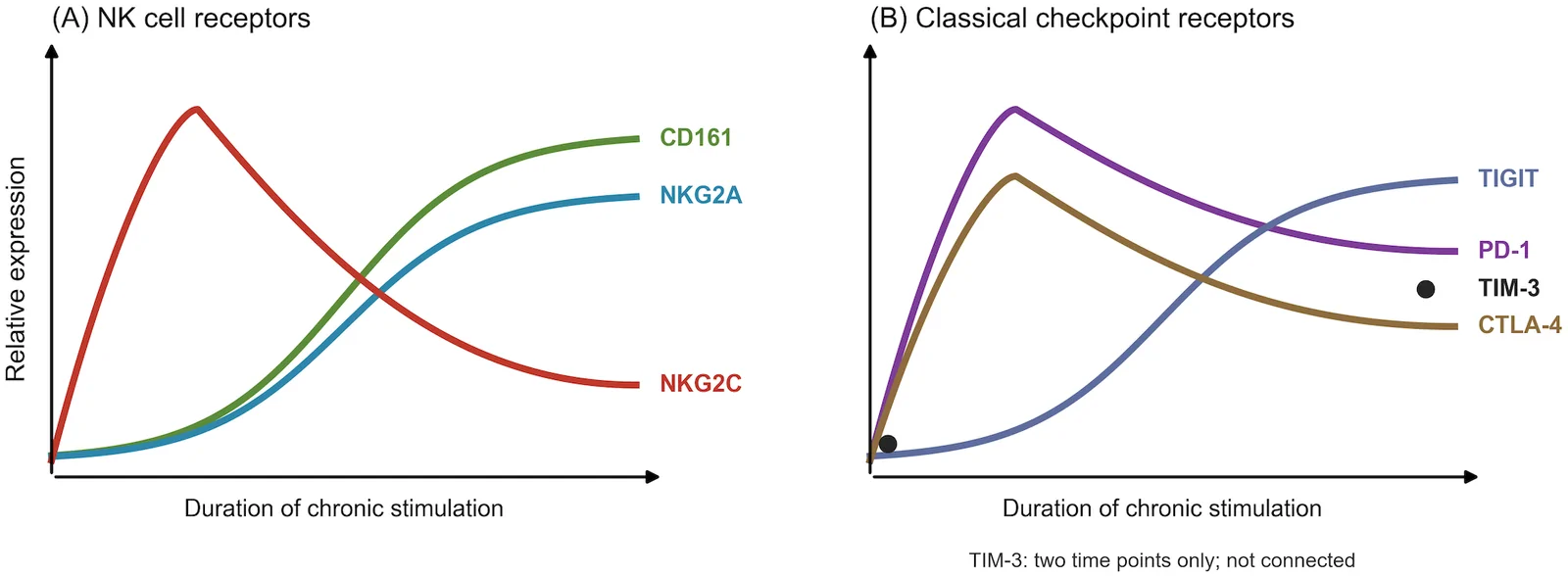
Protein-level dynamics of NK receptors and classical checkpoint receptors during chronic antigen stimulation. Schematic representation of surface protein dynamics during chronic antigen stimulation, based on *in vitro* CAR-T data (30) (Figure 4A, 1D; TIM-3 in Figure S1D of that study). Relative expression is plotted against stimulation duration. **(A)** NK cell receptors: Activating receptor NKG2C exhibits early transient upregulation followed by a decline, whereas inhibitory receptors NKG2A and CD161 (KLRB1) progressively increase. **(B)** Classical checkpoint receptors: PD-1 and CTLA-4 exhibit early transient upregulation followed by a decline to moderate levels (similar to NKG2C), whereas TIGIT progressively increases. TIM-3 is plotted as discrete points because only pre- and post-stimulation data are available. Validation in endogenous, non-engineered AENK-T cells remains required (Section 4.3).

The microenvironmental inputs that drive this accelerated transition operate through defined molecular cascades: metabolic constraints and suppressive cytokines feed directly into intracellular transcriptional networks (Section 4), while tumor-expressed ligands engage newly acquired inhibitory NKRs to enforce functional suppression (Section 5). The clinical implications of this accelerated temporal shift are discussed in detail in Section 6. Specifically, this section covers how the shift informs the optimal timing of therapeutic intervention.

## 4 Molecular mechanisms governing the dual-state transition

The transition across this temporal hierarchy involves complex intracellular regulation. This regulation determines whether a cell stabilizes as an Effector AENK-T or transitions into an Exhausted AENK-T (**Figure 4**).

**Figure 4:**
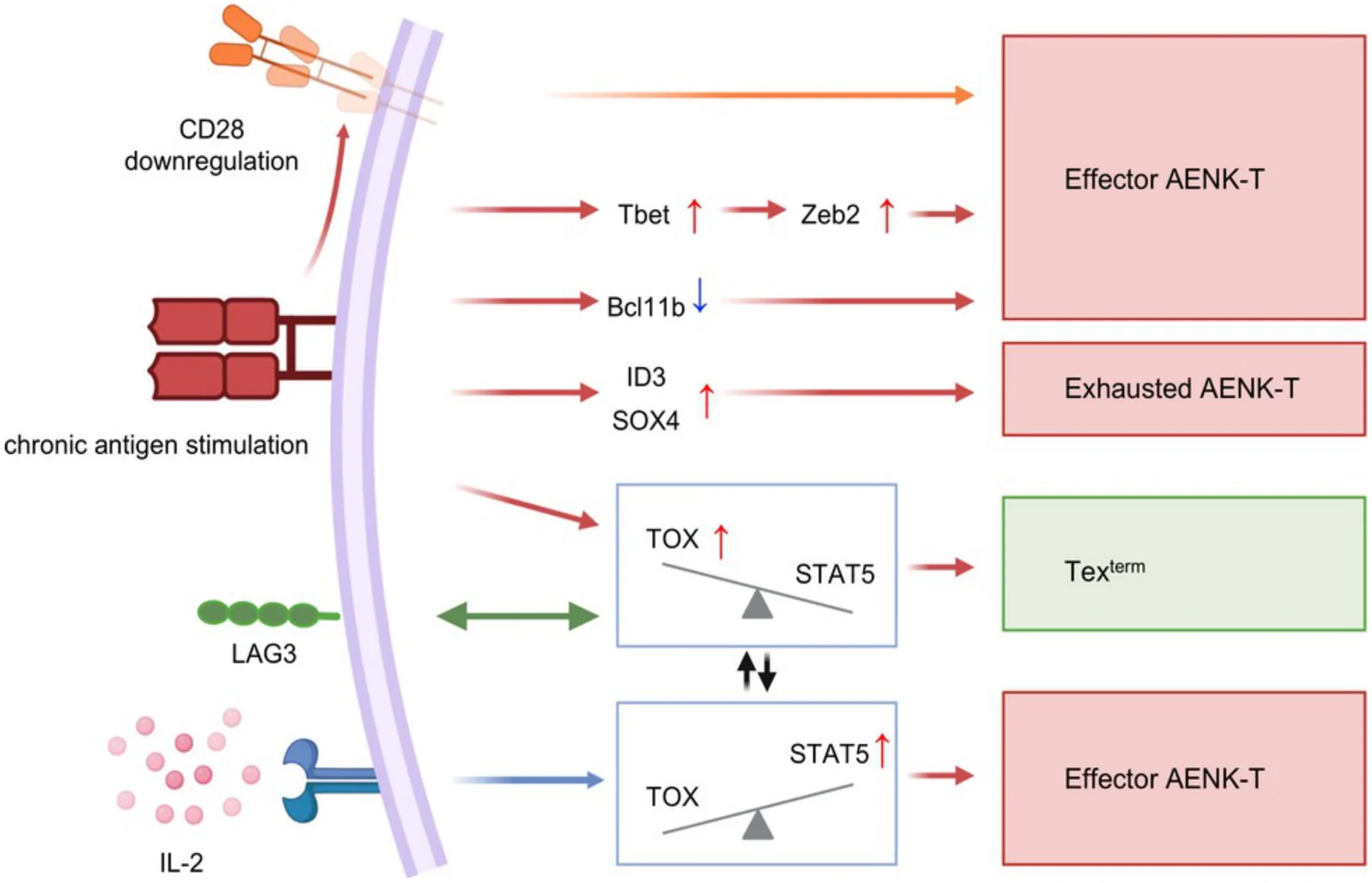
Molecular Mechanisms Governing AENK-T Cell Differentiation. Chronic antigen stimulation, accompanied by CD28 downregulation, engages a transcriptional network that operates within the T cell exhaustion program and directs cells toward distinct states. Downregulation of BCL11B de-represses NK-lineage genes, and the T-bet–Zeb2 axis promotes cytotoxicity and chromatin accessibility at loci encoding activating NK receptors; both favor the Effector AENK-T state. The SOX4–ID3 axis instead induces inhibitory NK receptors and drives the Exhausted AENK-T state. Persistent stimulation and microenvironmental stress activate TOX, and a positive feedback loop between TOX and LAG-3 sustains inhibitory NK receptor expression, reinforcing both the Exhausted AENK-T state and terminal exhaustion (Tex^term^). IL-2–STAT5 signaling opposes this by reducing chromatin accessibility at TOX enhancer regions, thereby limiting terminal exhaustion and favoring the Effector AENK-T state. Exhausted AENK-T and Tex^term^ cells are shown separately: the two states converge functionally, in that both lose effector function, but whether they converge phenotypically remains undetermined.

### 4.1 The requisite shift: CD28 downregulation and NKR acquisition

Persistent antigen stimulation triggers a progressive functional shift in T cells, characterized by the downregulation of the co-stimulatory molecule CD28. Transcriptomic and *in vitro* data suggest a mutually reinforcing feedback loop. As conventional CD28-dependent activation becomes impaired, T cells compensatorily upregulate NKRs to maintain functional competence. This upregulation, in turn, further facilitates the loss of CD28 (40, 41). While direct mechanism-level *in vivo* validation of this loop remains an active area of investigation, this fundamental shift sets the stage for further differentiation.

### 4.2 Transcriptional drivers of the effector AENK-T state

While optimal TCR avidity was initially hypothesized to solely determine the Effector state, it is now understood that a complex blend of specific sub-optimal avidity signals and localized microenvironmental cues biases the trajectory toward innate-like cytotoxicity (14, 42). Several key transcriptional networks drive this effector reprogramming. For instance, BCL11B maintains T cell identity by regulating intrinsic genes such as Notch1, Hes1, and Gata3. It also interacts with the DNA methyltransferase DNMT1 to ensure the DNA methylation fidelity of these genes (43). Persistent antigen stimulation downregulates BCL11B. This downregulation diminishes T cell-associated genes while upregulating NK lineage-related genes (e.g., NKG2C), thereby establishing the baseline Effector AENK-T differentiation (43, 44). Concurrently, IL-2/STAT5 signaling antagonizes the exhaustion-driving factor TOX by reducing chromatin accessibility at its enhancer regions (45). When TOX is suppressed and sufficient cytokine support is present, CD8+ T cells avoid terminal exhaustion and upregulate activating NKRs such as NKG2D. However, within the nutrient-depleted TIME, limited IL-2 availability restricts this protective STAT5 axis, lowering the threshold for TOX-driven exhaustion. Furthermore, sustained stimulation upregulates Zeb2 via T-bet. Zeb2 then closes chromatin at memory genes and opens it at loci associated with cytotoxicity and activating NKRs (15).

### 4.3 Transcriptional drivers of the exhausted AENK-T state

Conversely, continuous antigen stimulation and TIME microenvironmental stress activate networks that drive cells toward the Exhausted AENK-T state. Metabolic constraints within the tumor core act as primary catalysts: oxygen tension falls below physiological levels, and infiltrating T cells progressively lose mitochondrial mass and function via repression of biogenesis (46). Severe glucose competition and phosphoenolpyruvate depletion blunt TCR-driven calcium signaling (47), while lactate accumulation and acidosis add further suppressive pressure (27). In the absence of robust IL-2/STAT5 antagonism (Section 4.2), chronic TCR engagement continuously drives NFAT-dependent signaling without its primary counterweight, allowing TOX-driven programs to prevail.

At the core of this dysfunctional transition is TOX, the primary transcription factor driving terminal exhaustion (Tex^term^). TOX and the immune checkpoint receptor LAG-3 form a critical positive feedback loop. TOX directly induces LAG-3 expression; conversely, sustained LAG-3 signaling maintains high TOX levels. This continuous LAG-3 signaling actively suppresses beneficial NK-like effector reprogramming. By stabilizing the core exhaustion pathway, this loop reinforces Tex^term^ while suppressing functional NK-like effector differentiation (48).

In addition to the TOX/LAG-3 axis, recent insights (derived predominantly from CAR-T cell models) have identified another layer of transcriptional regulation involving SOX4 and ID3. In these engineered cells, SOX4 directly binds to regulatory elements at inhibitory NKR loci, while ID3 facilitates this dysfunctional transition through protein-protein interactions (30, 49). Although the SOX4/ID3 axis represents a potential collaborative mechanism, these findings require further validation in endogenous, non-engineered AENK-T cells responding to natural microenvironmental pressures.

## 5 Functional competence: distinct roles of effector and exhausted AENK-T cells

The phenotypic acquisition of NKRs translates directly into capabilities operating beyond conventional TCR signaling. The immunological output heavily depends on the cell’s differentiation state and the temporal hierarchy. The following section provides an integrated overview of the key NKRs and classical checkpoint receptors regulated in AENK-T cells, organized by receptor/molecule type, ligand, associated differentiation state, temporal expression window, clinical prognostic significance, and supporting primary studies (**Table 1**).

### 5.1 “Effector AENK-T cells”: innate-like cytotoxicity via activating NKRs

Effector AENK-T cells acquire potent cytotoxic capabilities through activating NKRs, providing important defense against tumors that downregulate MHC class I molecules.

#### 5.1.1 NKG2D

NKG2D recognizes stress-induced molecules (MICA/B) on tumor cells (50). It provides important co-stimulation for CD28-negative cells and enables TCR-independent killing of target cells (51). Clinically, high NKG2D expression on TILs is a favorable prognostic factor in many solid tumors, including melanoma and gastric cancer (32, 33). In colorectal cancer, high expression of NKG2D ligands on tumor cells, particularly MIC and RAET1G, is similarly associated with prolonged patient survival, a finding consistent with immunoediting pressure that progressively selects for ligand-low tumor clones (31). This expression correlates with potent antitumor activity and improved overall survival (52). However, persistent NKG2D signaling in the TIME can eventually contribute to exhaustion. Tumors also actively counteract this activating arm through microenvironmental suppressive factors. Specifically, elevated TIME levels of TGF-β potently downregulate NKG2D on CD8+ T cells, impairing their cytotoxic capacity (53, 54). This loss of activating signaling occurs alongside active tumor evasion via NKG2D ligand shedding (55), selectively stripping Effector AENK-T cells of their functional advantage while inhibitory NKRs accumulate.

#### 5.1.2 NKG2C

NKG2C (paired with CD94) is an activating NK receptor rapidly induced during the early Effector phase. It specifically recognizes HLA-E, a non-classical MHC class I molecule frequently upregulated on stressed or virally infected cells. A defining feature of NKG2C+ AENK-T cells is the dual mobilization of cytotoxic granules (granzymes and perforin). These proteins can be released through conventional TCR signaling or, importantly, through TCR-independent mechanisms triggered by HLA-E engagement (24, 34, 56). The latter is mediated via the adaptor protein DAP12, which couples NKG2C ligation to downstream cytotoxic signaling independently of TCR activation (9, 24). This dual-release capacity endows Effector AENK-T cells with the ability to eliminate MHC class I-downregulated tumor cells that would otherwise evade conventional TCR-dependent cytotoxicity.

### 5.2 “Exhausted AENK-T cells”: immune suppression via inhibitory NKRs

As defined by the temporal hierarchy, Exhausted AENK-T cells accumulate inhibitory NKRs during the later stages of chronic stimulation. Crucially, the TIME provides abundant cognate ligands for these late-emerging receptors, including HLA-E, HLA-C, and CLEC2D expressed on tumor and stromal cells, ensuring that inhibitory NKRs (NKG2A, KIR2DL, KLRB1) are potently engaged as soon as they appear, establishing robust tumor tolerance.

Inhibitory NKRs are not acquired in isolation. In chronically stimulated CD8+ T cells, they accumulate together with classical checkpoint receptors: while PD-1 and CTLA-4 remain co-expressed at sustained levels after their early induction, TIGIT rises in parallel with NKG2A as cells leave the Effector AENK-T state (30). The temporal hierarchy therefore describes one layer of a broader inhibitory program rather than a separate axis.

#### 5.2.1 NKG2A

NKG2A/CD94 progressively upregulates during late-stage chronic stimulation. NKG2A binds to the same ligand (HLA-E) as the activating NKG2C receptor (57). This receptor switch represents the core of the temporal hierarchy. Surface plasmon resonance measurements with soluble receptors show that the inhibitory CD94/NKG2A receptor binds HLA-E with a higher affinity than the activating CD94/NKG2C receptor, and that recognition by both is peptide dependent (58). Consequently, as TOX drives the progressive transcriptional upregulation of NKG2A during chronic stimulation, it outcompetes NKG2C for HLA-E engagement. By subsequently recruiting SHP-1/SHP-2 phosphatases via ITIM domains, NKG2A inhibits TCR and activating NKR signals. This shift from DAP12-mediated activation to dominant suppression facilitates tumor evasion (57). Prognostically, high co-expression of NKG2A and HLA-E functions as an independent poor prognostic factor in ovarian cancer and non-small cell lung cancer (NSCLC). This synergy directly attenuates CTL efficacy and shortens overall survival (35, 36).

#### 5.2.2 Inhibitory KIRs (KIR2DL)

Sustained antigen stimulation induces Killer-cell immunoglobulin-like receptors (KIRs), which facilitate CD28 downregulation and serve as an adaptive process to avoid excessive activation. It is important to distinguish between activating KIRs (e.g., KIR2DS) and inhibitory KIRs (e.g., KIR2DL1, KIR2DL2). In the exhausted state, inhibitory KIRs selectively dominate (59). KIR2DL2 demonstrates precise spatiotemporal control. Following ligand binding, it localizes to the synapse periphery without initially inhibiting cytotoxicity. After 15–30 minutes, it translocates to the central synapse and selectively suppresses the phosphorylation of PLC-γ1, Vav1, and ERK1/2. This preserves TCR-dependent cytotoxic capacity while selectively suppressing inflammatory functions like IFN-γ production and proliferation (60).

Recent studies have revealed that KIR+ CD8+ T cells represent a specialized regulatory subset (37, 61). Under physiological conditions, they serve as a homeostatic safeguard. They selectively eliminate aberrantly activated pathogenic CD4+ T cells via their preserved cytotoxic effector function, while KIR signaling simultaneously suppresses excessive systemic inflammation. In cancer, these KIR+ AENK-T cells function as Treg-like immunosuppressive subsets. The expression of inhibitory KIR2DL2, especially in the presence of HLA-C1 ligands, promotes robust immune evasion and correlates strongly with poor patient prognosis (37).

#### 5.2.3 KLRB1 (CD161)

In chronic tumor stimulation, KLRB1 primarily functions as a co-inhibitory receptor. Its ligand, CLEC2D (LLT1), is frequently expressed on tumor cells. Upon engaging this ligand, KLRB1 signaling significantly blunts cytokine production and cytotoxic activity (38). This presents a paradox: KLRB1 is often a favorable prognostic factor, yet it transmits inhibitory signals. This discrepancy is resolved by understanding that in the absence of CLEC2D, KLRB1 expression acts merely as a surrogate marker for prior activation and a highly cytotoxic state. The receptor’s inhibitory function manifests only when it engages CLEC2D. Thus, in malignant gliomas and hematological malignancies, where tumors actively upregulate CLEC2D, the result is profound T cell suppression and shortened survival (39). Consequently, targeting KLRB1 therapeutically requires strict patient stratification. Pre-treatment biopsies must confirm CLEC2D overexpression to ensure the receptor is acting in an inhibitory capacity.

## 6 Therapeutic implications: harnessing AENK-T cells for cancer immunotherapy

Immune checkpoint blockade (ICB), which releases the inhibitory brakes on T cells, has produced long-lasting responses across a range of tumor types (62); however, given the high rates of primary refractoriness (63), identifying novel therapeutic targets is a priority. Inhibitory NKRs, which are frequently co-expressed with PD-1 on tumor-infiltrating CD8+ T cells, represent promising next-generation ICB candidates.

For example, HLA-E is overexpressed in many tumors and potently suppresses AENK-T cell activity via NKG2A. Clinical trials of the anti-NKG2A antibody monalizumab have provided important lessons. In advanced, metastatic solid tumors, combination therapy (monalizumab + PD-L1 blockade) yielded limited objective response rates (64). In contrast, early-stage trials (NeoCOAST and COAST in non-small cell lung cancer) demonstrated substantial improvements in major pathological response and progression-free survival (65, 66). This presents an apparent paradox: if NKG2A is a late-stage marker of the terminal Exhausted AENK-T state, why does early intervention work best?

Early clinical targeting of NKG2A may prevent the transition into irreversible exhaustion by intervening during the plastic phase, which occurs after upregulation begins but before epigenetic changes become rigid. While this is a compelling mechanism, these findings must also account for potential confounding factors. For instance, differences in disease stage, tumor burden, and PD-L1 expression across trials cannot be entirely excluded.

Early intervention also carries a trade-off that the same framework anticipates. Unlike the progressive accumulation of inhibitory NKRs under sustained chronic stimulation depicted in **Figure 3** and **Table 1**, inhibitory NKRs in physiological contexts are moderately induced above naive levels during the acute phase of a resolving infection and persist at homeostatic levels thereafter, placing them within the normal regulation of antigen-experienced CD8+ T cells rather than outside it. NKG2A is not only a brake: during acute viral infection, it is required to sustain virus-specific CD8+ T cells and for host resistance (67). The framework predicts that early NKG2A blockade affects three homeostatic functions: restraint of cytotoxicity toward normal tissue that expresses HLA-E, protection of the Effector AENK-T pool against activation-induced cell death under unopposed stimulation, and inhibition of NK cells, which use the same receptor for missing-self surveillance. Clinical data do not yet show this trade-off, since neither monalizumab monotherapy nor its combination with durvalumab produced severe autoimmune toxicity in NeoCOAST or COAST (65, 66). This model would predict that the discrepancy reflects the restricted distribution of HLA-E, which is upregulated on tumors and at inflamed sites rather than across normal tissue, so that a systemic antibody acts mainly where its ligand is concentrated. Longer follow-up in early-stage disease would test this.

Meanwhile, KLRB1 (CD161) therapy underscores the importance of a precision biomarker approach. Because KLRB1 relies on engaging CLEC2D (LLT1), the success of anti-KLRB1 ICB requires a strict patient stratification strategy relying on pre-treatment biopsies to confirm abundant CLEC2D tumor expression, ensuring the receptor functions in an inhibitory capacity (39).

“Armoring” strategies that manipulate transcription factor networks in CAR-T cells directly reflect the two-state duality of AENK-T cells. This engineering actively promotes beneficial Effector AENK-T traits while obstructing pathways leading to Exhausted AENK-T cells. To prevent exhaustion, we previously engineered CAR-T cells with a double knockout (DKO) of ID3 and SOX4. These transcription factors normally amplify the inhibitory NKR program; their removal prevented tumor recurrence *in vivo* and improved long-term survival (49). Conversely, other approaches confer Effector traits via CRISPR-Cas9 knockout of BCL11B or DNMT1 inhibition. These modifications generate highly cytotoxic Effector AENK-T CAR-T cells loaded with activating NKRs (43, 68). To extend this concept, researchers have engineered T cells with chimeric receptors that incorporate the activating NKG2D receptor and DAP10/12 signaling domains (69).

Whether inhibitory NKRs should be removed from CD8+ T cells requires careful consideration, since NK cells carry the same receptors. The relevant distinction is between systemic antibody blockade and cell-intrinsic knockout in a transferred product. An antibody reaches NK cells as well as T cells and can disturb missing-self surveillance, because NKG2A and inhibitory KIRs are the receptors through which NK cells are licensed to spare normal cells that express HLA class I. A knockout is confined to the infused cells and leaves endogenous NK cell education intact. The ID3/SOX4 DKO CAR-T design follows this logic and goes one step further, since it removes upstream transcription factors rather than a single receptor and thereby lowers the inhibitory NKR program as a whole (49). The coordinated upregulation and potential functional overlap of inhibitory receptors make this distinction practically important. Inhibitory NKRs, including NKG2A, inhibitory KIRs, and KLRB1, as well as TIGIT are progressively upregulated in association with sustained PD-1 co-expression (Section 5.2). The framework predicts that knockout of a single receptor yields limited benefit, and that control at the transcription factor level, or simultaneous targeting of several receptors, is required.

Future therapeutic strategies could bypass traditional TCR limitations by integrating three key approaches: early-stage NKG2A blockade, biomarker-driven patient selection (e.g., CLEC2D screening), and advanced effector-skewed CAR-T cell engineering. Together, these methods may actively leverage the innate-like cytotoxicity of true Effector AENK-T cells to overcome malignant evasion, provided that the diverted cells retain the capacity to enter the tumor.

## 7 Conclusion and future perspectives

In conclusion, AENK-T cells represent a dynamic trajectory characterized by dual-state plasticity. Driven by transcription factor reprogramming (e.g., BCL11B, TOX, and SOX4) and microenvironmental cues, these cells bifurcate into two paths. They either become highly potent Effector AENK-T cells armed with activating NKRs (NKG2C, NKG2D), or transition into deeply suppressive Exhausted AENK-T cells. The latter state is dominated by inhibitory NKRs (NKG2A, KIRs, KLRB1) and overlaps with classical terminal exhaustion. While physiological contexts like infection utilize the temporal hierarchy for balanced host defense, the TIME drives these cells into a terminal state, exploiting inhibitory NKRs for immune evasion.

Beyond summarizing these observations, the temporal hierarchy generates predictions that current methods can test. First, the framework predicts that the benefit of NKG2A blockade is confined to the plastic window that opens once NKG2A is expressed and closes as the exhaustion program becomes epigenetically fixed. Efficacy should therefore be largest in early-stage or neoadjuvant settings and small in advanced disease, which is the pattern seen when NeoCOAST and COAST are set against the advanced solid tumor trial (64–66). A prospective trial stratified by stage within a single tumor type, or an analysis relating the pre-treatment NKG2A^+^ fraction to response, would test it. Second, the framework predicts that patients whose tumor-infiltrating CD8+ T cells express NKG2A alongside high NKG2C, or a TOX-low signature, hold a responsive Effector-like AENK-T compartment and gain the most from NKG2A blockade, whereas those with a TOX-high signature derive limited benefit. Single-cell and flow cytometry data from completed ICB trials allow this to be tested retrospectively. Third, the framework predicts that the benefit of anti-KLRB1 therapy is conditional on tumor CLEC2D expression, and that KLRB1 marks cytotoxic capacity without therapeutic value in CLEC2D-negative tumors. Stratification by CLEC2D expression on pre-treatment biopsies would test it. Each prediction is falsifiable with data of the kind already generated in ongoing trials, which is what distinguishes the temporal hierarchy from a descriptive summary.
